# K63-Linked Polyubiquitination on TRAF6 Regulates LPS-Mediated MAPK Activation, Cytokine Production, and Bacterial Clearance in Toll-Like Receptor 7/8 Primed Murine Macrophages

**DOI:** 10.3389/fimmu.2018.00279

**Published:** 2018-02-21

**Authors:** Jaya Talreja, Lobelia Samavati

**Affiliations:** ^1^Department of Internal Medicine, Division of Pulmonary, Critical Care and Sleep Medicine, Wayne State University School of Medicine, Detroit Medical Center, Detroit, MI, United States; ^2^Center for Molecular Medicine and Genetics, Wayne State University School of Medicine, Detroit, MI, United States

**Keywords:** toll-like receptor 7/8, toll-like receptor 2, toll-like receptor 4, ubiquitination, priming, bone marrow-derived macrophages, post viral–bacterial infection

## Abstract

Post viral infection bacterial pneumonia is a major cause of morbidity and mortality associated with both seasonal and pandemic influenza virus illness. Despite much efforts put into the discovery of mechanisms of post viral–bacterial infections and their complications in recent years, the molecular mechanisms underlying the increased susceptibility to bacterial infection remain poorly understood. In this study, we focused on the pathways regulating immune responses in murine macrophages and modeled post viral–bacterial infections through pretreatment of bone marrow-derived macrophages (BMDMs) with a toll-like receptor (TLR) 7/8 ligand (R848) and subsequent challenge with TLR2/4 agonists to mimic bacterial infection. We found R848-primed BMDMs upon subsequent exposure to TLR2/4 ligands respond with enhanced inflammatory cytokine production, especially IL-6 and TNF-α. The enhanced cytokine production in R848-primed BMDMs in response to TLR2/4 was due to increased TGF-β-activated kinase (TAK) 1 phosphorylation with subsequent activation of ERK and p38 MAPKs. Furthermore, we identified that R848 priming leads to increased K63-linked polyubiquitination on TRAF6. K63-linked polyubiquitination on TRAF6 is a signal leading to enhanced activation of downstream pathways including TAK1. Importantly, R848-primed BMDMs infected with live bacteria exhibited decreased bacterial clearance. Small-molecule enhancer of rapamycin 3, an ubiquitin ligase inhibitor reversed the K63-linked polyubiquitination on TRAF6 in R848-primed BMDMs and subsequently decreased TAK1 and MAPK phosphorylation, and cytokine production as well as reversed the decreased bacterial clearance capacity of BMDMs. Our study may provide a novel molecular target to alleviate post viral–bacterial infections.

## Introduction

Viral infections predispose the host to secondary bacterial infections (1). Post-influenza bacterial pneumonia is a major cause of morbidity and mortality associated with both seasonal and pandemic influenza virus illness (2). Epidemiologic evidence suggests that mortality during the 1918–19 pandemic was mostly due to bacterial pneumonia (3). Several mechanisms have been postulated for the immunopathology of post viral–bacterial pneumonia. These mechanisms include bacterial colonization due to loss of mucociliary clearance, inhibition of the Th17 pathway, increased IL-10 production, suppression of macrophage function due to inhibition of NADPH oxidase dependent decreased phagocytic bacterial clearance (4, 5). The molecular mechanisms for such increased susceptibility to bacterial infections after a viral infection are not well understood. The cytokine storm triggered by post viral–bacterial infections may contribute to the mortality seen in viral infections, especially influenza infection (6).

Toll-like receptors (TLRs) are type I transmembrane proteins that mediate the recognition of pathogen-associated molecular patterns (7). TLR7/8 receptors are located in the intracellular endosomal compartment and recognize single-stranded RNA and imidazoquinolines (8). Except TLR3, engagement of all other TLRs recruits adaptor protein MyD88. While TLR3 is only TRIF dependent, TLR4 signaling is both MyD88 and TRIF dependent (9). Recruitment of adaptor proteins activates IL-1 receptor-associated kinases (IRAK) 4 and IRAK2 (8). Activation of IRAKs results in an interaction with TRAF6, an E3 ligase, to trigger lysine 63 (K63) auto-polyubiquitination of TRAF6. Polyubiquitinated TRAF6 on K63 forms a recognition signal for the recruitment of TGF-β-activated kinase (TAK) 1 binding protein (TAB) 2/3 to activate TAK1. Subsequent TAK1 activation leads to NF-κB and MAPK activation resulting in the production of inflammatory cytokines (7, 10, 11).

In this study, we modeled the effect of a viral infection through priming of bone marrow-derived macrophages (BMDMs) with a TLR7/8 ligand (R848) and subsequent activation of the primed BMDMs with TLR2 or TLR4 ligands. We found that priming of BMDMs with R848 enhances the inflammatory cytokine production in subsequent activation with either TLR2 or TLR4 ligands. Enhanced cytokine production by R848-primed BMDMs in response to TLR2/4 was due to increased activation and phosphorylation of TRAF6 and TAK1, with a subsequent enhanced activation of MAPKs. Furthermore, our study shows that R848 priming leads to increased K63-linked polyubiquitination on TRAF6. Interestingly, we found that an inhibitor of the ubiquitin ligase downregulated the expression of TRAF6 and inhibited TAK1 and MAPKs phosphorylation with a subsequent reduction of pro-inflammatory cytokine production in R848-primed BMDMs in response to LPS. In addition, exposure of R848-primed BMDMs to live *E. coli* bacteria yielded a decreased clearance of live bacteria but increased cytokine production. This effect was reversed in the presence of the inhibitor of the ubiquitin ligase.

## Materials and Methods

### Chemicals

All ligands, LPS, Pam3CSK4 (PAM), and R848, were purchased from InvivoGen (San Diego, CA, USA). Ubiquitin ligase inhibitor (ULI) small-molecule enhancer of rapamycin (SMER) 3 was purchased from Selleckchem (Houston, TX, USA). Protein A-Sepharose beads were purchased from Sigma (St. Louis, MO, USA). Phospho-specific antibodies against ERK1/2, p38, c-Jun NH(2)-terminal kinase (JNK), TAK1, as well as TRAF6, K63-specific polyubiquitin and β-actin were purchased from Cell Signaling Technology (Beverly, MA, USA). Antibodies against p38 and polyubiquitin were purchased from Santa Cruz Biotechnology (Santa Cruz, CA, USA). Horseradish peroxidase (HRP)-conjugated anti-mouse and anti-rabbit IgG secondary antibodies were purchased from Cell Signaling Technology, and HRP-conjugated anti-goat antibody was purchased from Santa Cruz Biotechnology (Santa Cruz, CA, USA), and FITC-labeled anti-CD11b was purchased from BD Biosciences (San Jose, CA, USA).

### Mice and Isolation of BMDMs

C57BL6 wild-type mice were used to isolate BMDMs. Animal studies were approved by the University Committees on Use and Care of Animals. BMDMs from mice were prepared as described previously (12). Briefly, femurs and tibias from 6- to 12-week-old mice were dissected, and the bone marrow was flushed out. Cells were cultured with IMDM media supplemented with 30% L929 conditioned media, glutamine, sodium pyruvate, 10% heat-inactivated fetal FBS, and antibiotics for 5–7 days (13). BMDMs were re-plated at a density of 2 × 10^6^ cells/well the day before each experiment. Under this standardized method, the purity of BMDMs was 99% as assessed by the presence of CD11b using flow cytometry (Figure S1 in Supplementary Material).

### Flow Cytometry and Cell Surface Immunostaining

To assess the purity of BMDMs, cells were stained for the cell surface marker using fluorescent labeled antibody for CD11b. Flow cytometry was performed on a BD LSR II SORP, and data analysis was performed using FlowJo software (FlowJo, LLC, Ashland, OR, USA). Samples were gated on cells using FSC/SSC. The flow cytometry work was done at Microscopy, Imaging and Cytometry Resources Core at The Karmanos Cancer Institute, Wayne State University.

### R848 Priming of BMDMs and Subsequent Challenge with TLR4 (LPS) or TLR2 (PAM) Ligands

Bone marrow-derived macrophages were cultured in 6-well plates at a density of 2 × 10^6^ cells/well. The cells were stimulated with the TLR7/8 ligand R848 (100 ng/mL) for 24 h. The next day, cells were washed with PBS and challenged with the LPS (500 ng/mL) or PAM (500 ng/mL) for further 24 h for cytokine measurement or for different time periods for immunoblotting. To study the effect of ULI, SMER3, BMDMs were pretreated with R848 (100 ng/mL) and SMER3 (5 µm) for 24 h. The next day, cells were washed with PBS and challenged with LPS (100 ng/mL) for different time periods. SMER3 was solubilized in DMSO, and as a control BMDMs were treated with solvent alone in all the experiments.

### Protein Extraction and Immunoblotting

After the appropriate treatments, cells were washed with PBS and harvested in RIPA buffer (Millipore, MA, USA) containing protease inhibitor and anti-phosphatase cocktails, as previously described (14). Equal amounts of proteins were mixed with the same volume of 2× sample buffer, separated on 10% SDS-polyacrylamide gel electrophoresis and transferred to a polyvinylidene di-fluoride (PVDF) membrane (Bio-Rad, Hercules, CA, USA) at 20 V for 60 min using a semi dry transfer cell (Bio-Rad) as previously described (14). The PVDF membrane was blocked with 5% dry milk in Tris-buffered saline with 0.1% Tween-20, rinsed, and incubated with primary antibody overnight. The blots were washed and incubated with HRP-conjugated secondary anti-IgG antibody. Membranes were washed and immunoreactive bands were visualized using a chemiluminescent substrate (ECL-Plus, GE Healthcare, Pittsburgh, PA, USA) (15, 16). Images were captured on Hyblot CL film (Denville, Scientific Inc., Metuchen, NJ, USA). Optical density analysis of signals was performed using ImageQuant software (version 5, Molecular Dynamics).

### Enzyme-Linked Immunosorbent Assay (ELISA)

The cytokine levels of TNF-α, IL-6, IL-1β, and IL-10 were measured in cell culture supernatants using ELISA DuoKits (R&D Systems) as previously described (17).

### Measurement of Nitric Oxide (NO) Production

Nitric oxide levels in culture supernatants were quantified by Griess Reaction Assay as previously described (12).

### Immunoprecipitation

Bone marrow-derived macrophages were lysed with RIPA buffer (Millipore, MA, USA) containing protease inhibitor and anti-phosphatase cocktails, and the lysate was centrifuged to pellet the cell debris. The resulting supernatant was then used for immunoprecipitation. The lysate was incubated with TRAF6 antibody with 0.1% BSA overnight at 4°C with gentle rotation. Protein A-Sepharose beads (30 µL) were added and incubated for 2 h at 4°C with gentle rotation. After centrifugation, immunoprecipitation pellets were washed five times using ice-cold wash buffer (50 mM Tris–Cl, pH 7.4, 5 mM EDTA, 300 mM NaCl, 0.1% Triton X-100, and 0.02% sodium azide) with a brief centrifugation each time in a refrigerated microcentrifuge. Samples from the pellets were resuspended in the loading buffer and were subjected to gel electrophoresis and then was blotted onto PVDF membranes (Bio-Rad, Hercules, CA, USA). Membranes were blocked and then incubated overnight with K63-specific polyubiquitinated antibody. The next day, membranes were washed and incubated with HRP-conjugated secondary antibody for 2 h at room temperature. Membranes were washed again, and signal was detected using a chemiluminescence reagent.

### Bacterial Infection

The *E. coli* DH5α strain (Invitrogen) was used. Single colonies were inoculated into 5 mL of Luria Bertani medium and grown overnight at 30°C with shaking. On the day of the infection, a 1/5 dilution of the overnight culture was prepared and allowed to grow at 37°C with shaking to *A*_600_ = 0.5, corresponding to 10^9^ CFU/mL. BMDMs were cultured in 6-well plates at a density of 2 × 10^6^ cells/well. The cells were stimulated with R848 (100 ng/mL) or R848 (100 ng/mL) and SMER3 (5 µm) for 24 h. The next day, cells were washed with PBS and challenged with the *E. coli* suspended in 100 µL of PBS so that the final MOI is 10:1 (bacteria:BMDMs). After 1 h of infection at 37°C, macrophages were washed twice with PBS and IMDM supplemented with 10% heat-inactivated serum and 100 µg/mL of gentamicin, 50 U/mL of each penicillin and streptomycin to limit the growth of extracellular bacteria. Culture supernatants and cell lysates were collected 3 h after infection. For bacterial clearance assay, after 3 h of infection BMDMs were lysed in PBS containing 0.2% TritonX-100, and a serial dilution of lysate was streaked in duplicate on LB agar plates and incubated overnight at 37°C. CFUs were counted the next day.

### Cell Viability

Cells equivalent to 1 × 10^4^/well were seeded in 96-well cell culture plate and incubated for 24 h with different treatments. Cell viability was assessed using MTT assay [3-(4,5)-dimethyl thiazol-2,5-diphenyl tetrazolium bromide] as described previously (17). Absorbance was measured at 550 nm (12).

### Statistical Analyses

Statistical analyses were performed using SPSS software, version 23.0 (SPSS Inc., Chicago, IL, USA). One way analysis of variance test and *post hoc* repeated measure comparisons (least significant difference) were performed to identify differences between groups. ELISA results were expressed as mean ± SEM. For all analyses, two-tailed *p* values of less than 0.05 were considered significant.

## Results

### R848 Priming of BMDMs Increases the Production of Inflammatory Mediators in Response to TLR4 (LPS) or TLR2 (PAM) Ligands

**Figure 1:**
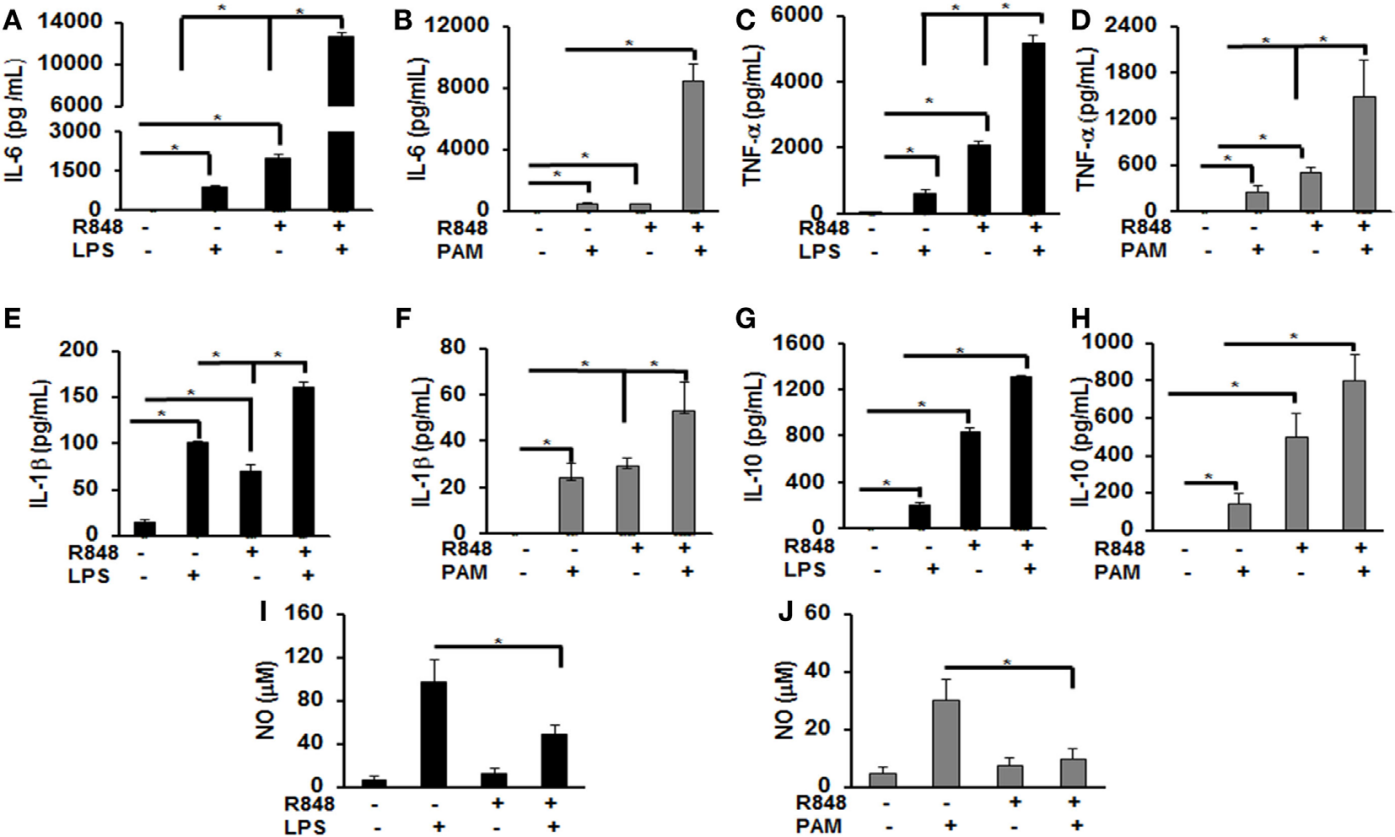
Increased production of inflammatory cytokines by R848-primed bone marrow-derived macrophages (BMDMs) in response to toll-like receptor (TLR) 4 (LPS) and TLR2 (PAM) ligands. Murine BMDMs were cultured at a density of 2 × 10^6^ cells/well and were preexposed to R848 (100 ng/mL) or medium alone for 24 h, washed and subsequently challenged with LPS (500 ng/mL) or PAM (500 ng/mL) for further 24 h. Conditioned media were analyzed for IL-6 **(A,B)**, TNF-α (**C,D**), IL-1β (**E,F**), and IL-10 (**G,H**) *via* enzyme-linked immunosorbent assay. Conditioned media were assayed for nitric oxide (NO) levels by nitrite quantification *via* Griess reaction **(I,J)**. Results shown are a mean of five independent experiments. A *p* value <0.05 was considered significant and error bars indicate SEM.

Previous studies identified several cytokines to play an important role in post viral–bacterial infections. Among those, three major cytokines playing a critical role in this process are IL-6, TNF-α, and IL-1β (18, 19). To examine the effect of priming murine BMDMs with a TLR7/8 ligand on their response to either LPS or PAM challenge, cells were treated with R848 (100 ng/mL) for 24 h. After the incubation periods, cells were washed twice, cultured in fresh medium and subsequently challenged with LPS (500 ng/mL) or PAM (500 ng/mL) for another 24 h. Murine IL-6, TNF-α, IL-1β, and IL-10 cytokine levels in the conditioned medium were measured *via* ELISA. As shown in Figure 1, primed BMDMs responded to LPS and PAM with a significantly higher IL-6 (Figures 1A,B), TNF-α (Figures 1C,D), IL-1β (Figures 1E,F), and IL-10 (Figures 1G,H) production as compared with unprimed BMDMs. In primed BMDMs, there was about 16-fold increase in IL-6 production and a 2-fold increase in IL-1β both with LPS or PAM treatment. An increase in TNF-α with LPS (ninefold) and PAM (sixfold) was observed. These results show that pre-exposure of macrophages to a TLR7/8 ligand (R848) leads to a significantly higher production of pro-inflammatory cytokines after subsequent challenge with either TLR2 or 4 ligands. Furthermore, we determined the NO production in the conditioned medium. There was a significant decrease in NO production (about 50%) in R848-primed BMDMs in response to LPS or PAM as compared with unprimed BMDMs activated with either LPS or PAM (Figures 1I,J). Data are presented as mean of five independent experiments and error bars indicate SEM.

### Primed BMDMs Respond to TLR4 and TLR2 Challenge with Distinct Increase of MAPK Activation

**Figure 2:**
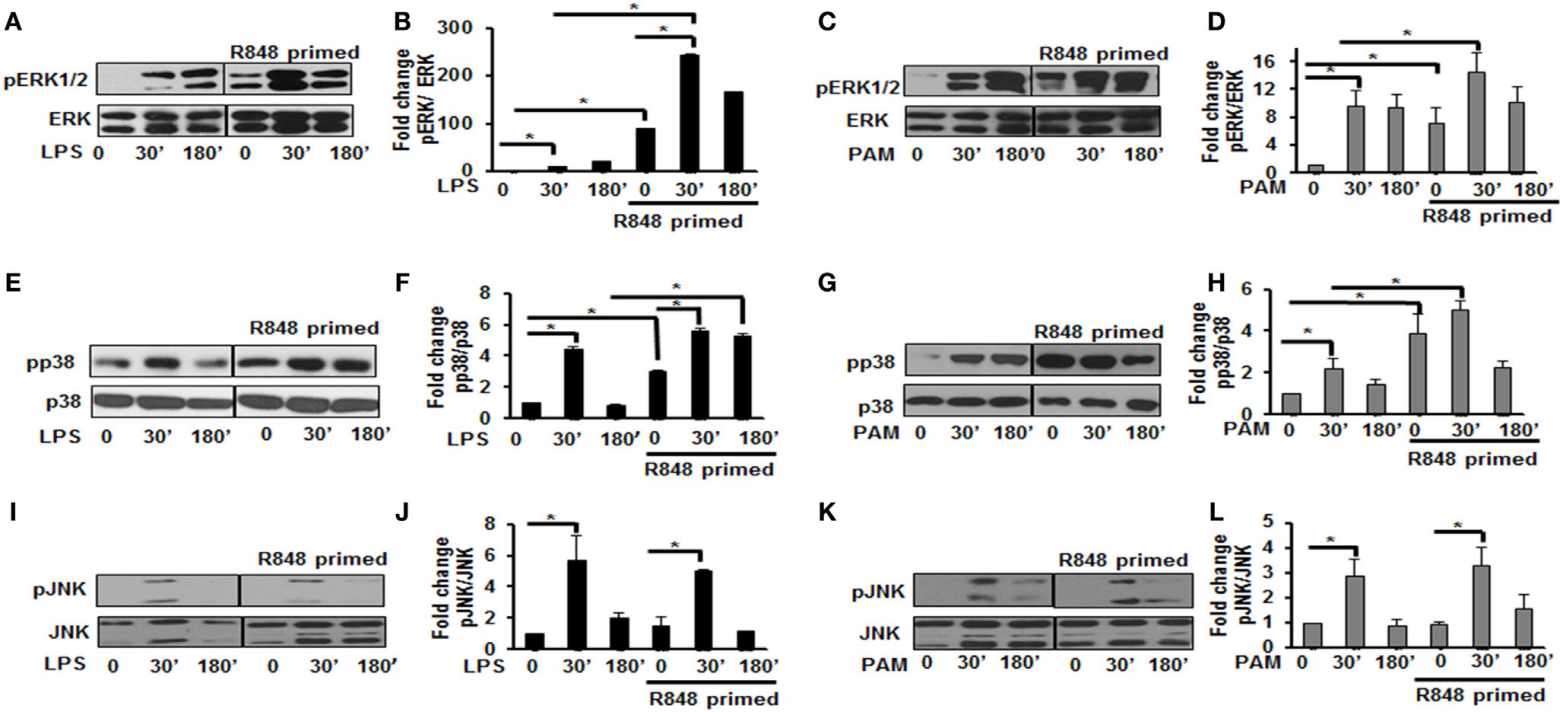
Distinct increase of MAPK phosphorylation in R848-primed bone marrow-derived macrophages (BMDMs). BMDMs were preexposed to R848 (100 ng/mL) or medium alone for 24 h, washed and subsequently challenged with LPS (500 ng/mL) or PAM (500 ng/mL) for different time periods as indicated. Whole cell extracts were prepared and subjected to SDS-PAGE and Western blot analysis using phosphorylated antibodies against ERK1/2, p38, and c-Jun NH(2)-terminal kinase (JNK). Equal loading was determined using antibodies against total ERK, total p38, and total JNK. Representative results of Western blots are shown out of a total of five independent experiments. Densitometry analysis is expressed as fold increase of the ratio phosphorylated protein/total protein. Expression of pERK in the absence or presence of LPS **(A,B)** and PAM **(C,D)**, expression of pp38 in the absence or presence of LPS **(E,F)** and PAM **(G,H)**, and expression of pJNK in the absence or presence of LPS **(I,J)** and PAM **(K,L)**. Densitometry results shown are mean of five independent experiments. A *p* value <0.05 was considered significant and error bars indicate SEM.

MAPKs are a family of serine threonine kinases that mediate signals in response to stress, microbial products, cytokines, and TLR ligands in various cell types (20). Several studies have shown that MAPKs, including extracellular signal regulated kinase (ERK), JNK, and the p38 kinase play important roles in regulating the responses to TLR activation and cytokine production. Therefore, we assessed the effect of priming with R848 (100 ng/mL) on the pattern of MAPKs activation. R848-primed or unprimed BMDMs were treated with LPS or PAM for different time periods and activation of ERK1/2, p38, and JNK was assessed by immunoblotting using antibodies against the phosphorylated forms of ERK (Thr202/Tyr204), p38 (Thr180/Tyr182), and JNK (Thr183/Tyr185). As shown in Figures 2A,B, primed cells responded to LPS stimulation with increased ERK1/2 phosphorylation. The mean densitometric values of the ratio pERK1/2/total ERK from five independent experiments are shown in Figure 2B. Activation of R848-primed BMDMs with PAM resulted in an increased phosphorylation of ERK1/2 at 30 min as compared with PAM alone (Figures 2C,D). Similarly, we found prolonged and sustained phosphorylation of p38 in primed BMDMs in response to LPS challenge as compared with unprimed cells (Figures 2E,F). The mean densitometric values of the ratio of pp38/p38 of five independent experiments are shown in Figure 2F. We observed similar results when the R848-primed BMDMs were challenged with PAM (Figures 2G,H). However, there was no difference in activation of JNK in response to either LPS or PAM in both primed and unprimed cells (Figures 2I–L). The mean densitometric values of the ratio pJNK/JNK from five independent experiments are shown in Figures 2J,L.

### R848-Primed BMDMs Respond to TLR4 and TLR2 Challenge with Increased Expression of TLR Signaling Molecules

**Figure 3:**
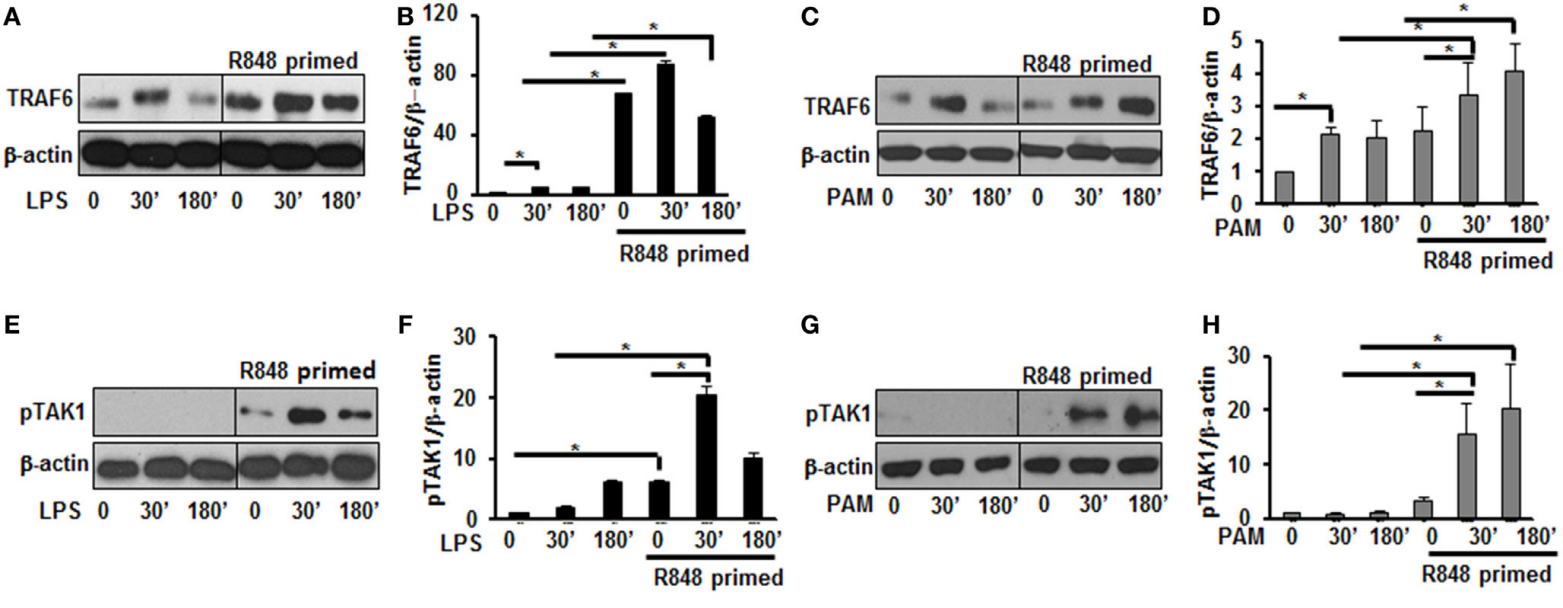
R848-primed bone marrow-derived macrophages (BMDMs) exhibit increased expression and activation of toll-like receptor signaling molecules. BMDMs were preexposed to R848 (100 ng/mL) or cultured in medium for 24 h, washed and subsequently challenged with LPS (500 ng/mL) or PAM (500 ng/mL) for different time periods. Whole cell extracts were prepared and subjected to SDS-PAGE and Western blot analysis using specific antibodies against TRAF6, pTAK1, and β-actin for equal loading was performed. Representative results of Western blots are shown out of a total of five independent experiments. Densitometry analysis expressed as fold increase of the ratio of specific protein/β-actin. TRAF6 in the absence or presence of LPS **(A,B)** and PAM **(C,D)**. Phosphorylated TGF-β-activated kinase 1 in the absence or presence of LPS **(E,F)** and PAM **(G,H)**. Densitometry results shown are mean of five independent experiments. A *p* value <0.05 was considered significant and error bars indicate SEM.

Engagement of TLRs by their ligands results in the activation and recruitment of several adaptor and signaling molecules that leads to the activation of MAPKs and NF-κB. Upon stimulation the adaptor molecule MyD88 recruits IRAKs to TLRs. IRAK-4, IRAK-1, and TRAF6 build a complex, which then interacts at the membrane with another kinase complex consisting of TAK1, TAB 1, and TAB 2 leading to the subsequent activation of MAPKs and NF-κB (21, 22). Since priming of BMDMs with R848 resulted in a significant production of pro-inflammatory cytokines and activation of MAPKs after subsequent TLR2/4 challenge, we determined the effect of priming on the expression and activation of molecules involved in TLR signaling. As shown in Figures 3A,B, R848-primed BMDMs exhibited increased expression of TRAF6 in response to LPS. The mean densitometric values of the ratio TRAF6/β-actin from five independent experiments are shown in Figure 3B. Similar results were obtained in response to PAM challenge (Figures 3C,D). Next, we determined the phosphorylated form of TAK1. As compared with unprimed cells R848-primed BMDMs exhibited increased expression of pTAK1 in response to LPS or PAM (Figures 3E–H). The mean densitometric values of the ratio pTAK1/β-actin from five independent experiments are shown in Figures 2F,H. These results indicate that priming of BMDMs with R848 increases the expression of TRAF6 and activates TAK1. Because we observed similar results when R848-primed BMDMs challenged with either LPS or PAM, to further elucidate the molecular mechanisms beyond the inflammatory hyperresponsiveness of R848-primed BMDMs, we performed further experiments with LPS.

### R848-Primed BMDMs Exhibit Enhanced K63-Linked Polyubiquitination

**Figure 4:**
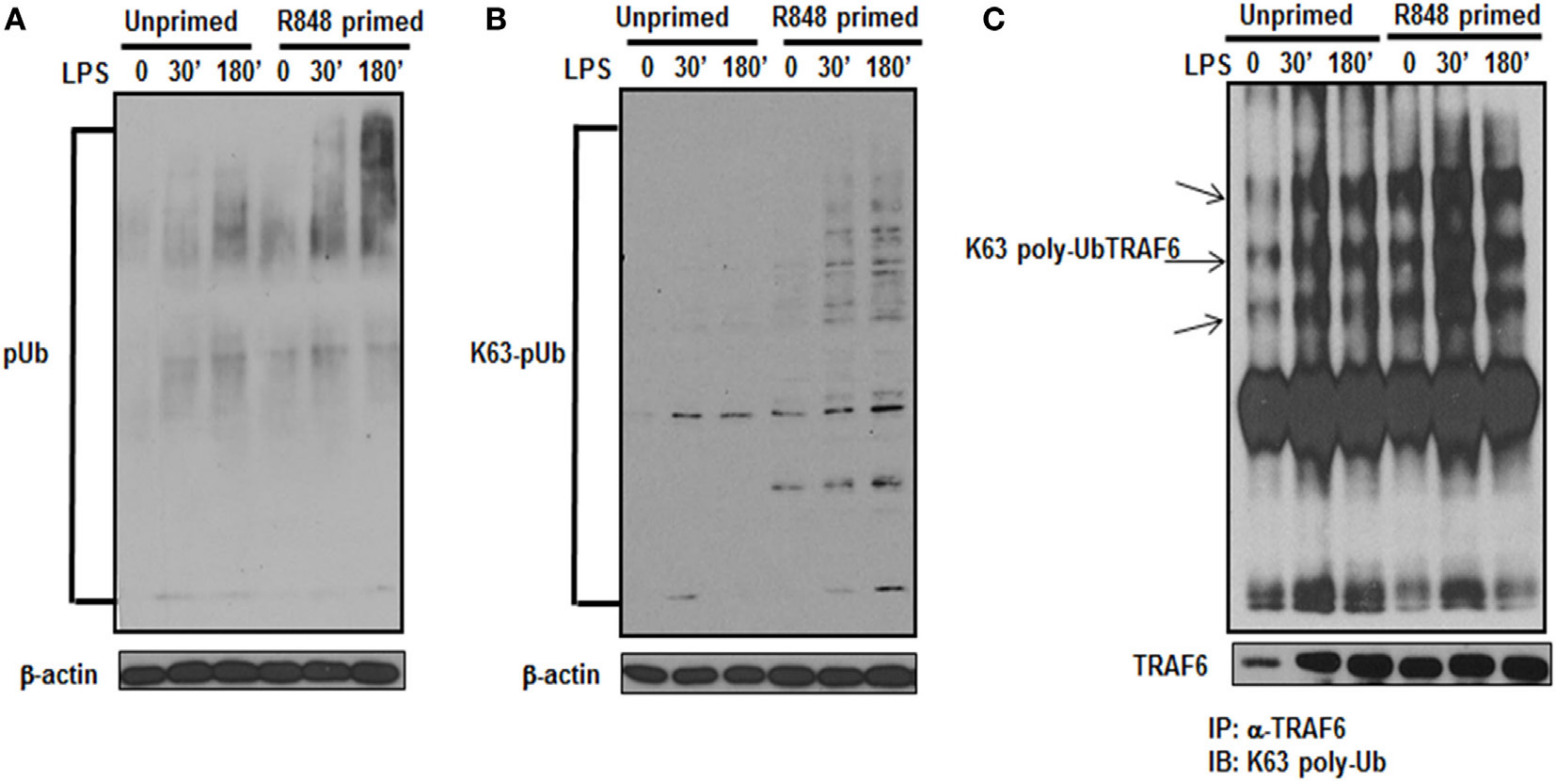
R848-primed bone marrow-derived macrophages (BMDMs) exhibit increased K63-specific polyubiquitination and upregulated expression of K63-linked polyubiquitinated TRAF6. BMDMs were preexposed to R848 (100 ng/mL) or medium alone for 24 h, washed and subsequently challenged with LPS (500 ng/mL) for different time periods as indicated. Whole cell extracts were subjected to SDS-PAGE, and Western blot analysis was performed using antibodies against polyubiquitin **(A)** or K63-linked polyubiquitin **(B)**. Representative results are shown out of a total of five independent experiments. Unprimed or R848-primed BMDMs were challenged with LPS (500 ng/mL) for 30 and 180 min. Cell lysates were prepared and immunoprecipitated (IP) for TRAF6 protein using TRAF6 antibody. IP protein was immunoblotted with K63-specific polyubiquitin antibody to detect K63-linked polyubiquitination on TRAF6 **(C)**. Representative result is shown out of a total of three independent experiments.

Activation of the TLR pathway is tightly regulated by ubiquitination. Lys-63 (K63)-linked ubiquitination is involved in phosphorylation and activation of TLR signaling pathways that leads to the activation of MAPKs and IkB kinases (23). To determine whether the increased activation of MAPKs and increased production of inflammatory cytokines in TLR7/8 primed cells is due to increased polyubiquitination, the level of polyubiquitinated proteins in primed and unprimed cells was assessed. R848-primed or unprimed BMDMs were cultured in the presence or absence of LPS for different time periods and cell lysates were assessed for polyubiquitination by immunoblotting using polyubiquitin and K63-specific polyubiquitin antibodies. As shown in Figure 4, R848-primed cells exhibit increased polyubiquitination (Figure 4A) as well as increased K63-specific polyubiquitination as compared with unprimed cells (Figure 4B). LPS challenge led to a further increase in expression of polyubiquitinated and K63-linked polyubiquitinated proteins. These data show that priming of BMDMs with a TLR7/8 ligand induces polyubiquitination specifically on K63 leading to increased activation of MAPKs and production of inflammatory cytokines by R848-primed cells in response to subsequent LPS challenge. Engagement of TLRs results in the activation of IRAK family members that interact with TRAF6, an E3 ligase, to trigger lysine 63 (K63) auto-polyubiquitination of TRAF6 and IRAK-1 (24). K63-linked polyubiquitinated TRAF6 forms a recognition signal for the recruitment of TAK1 binding protein 2/3 (TAB 2/3) to activate TAK1 (7, 25). Because R848 priming enhanced K63-linked polyubiquitinated proteins (Figure 4B), we determined the K63-linked polyubiquitination on TRAF6 by immunoprecipitation. Cell lysates were immunoprecipitated (IP) using TRAF6 antibody followed by immunoblotting using an anti-K63-linked polyubiquitin antibody. We found that R848-primed BMDMs exhibit increased expression of K63-linked polyubiquitinated TRAF6 as compared with unprimed cells (Figure 4C). Furthermore, activation of primed cells with LPS resulted in enhanced K63-linked polyubiquitination on TRAF6. These results indicate that R848 priming upregulates K63-linked polyubiquitination on TRAF6, which may result in increased activation of MAPKs and increased production of inflammatory cytokines in R848- primed cells in response to LPS.

### An E3 ULI Prevents R848-Primed Hyperactivation of MAPKs in Response to LPS

**Figure 5:**
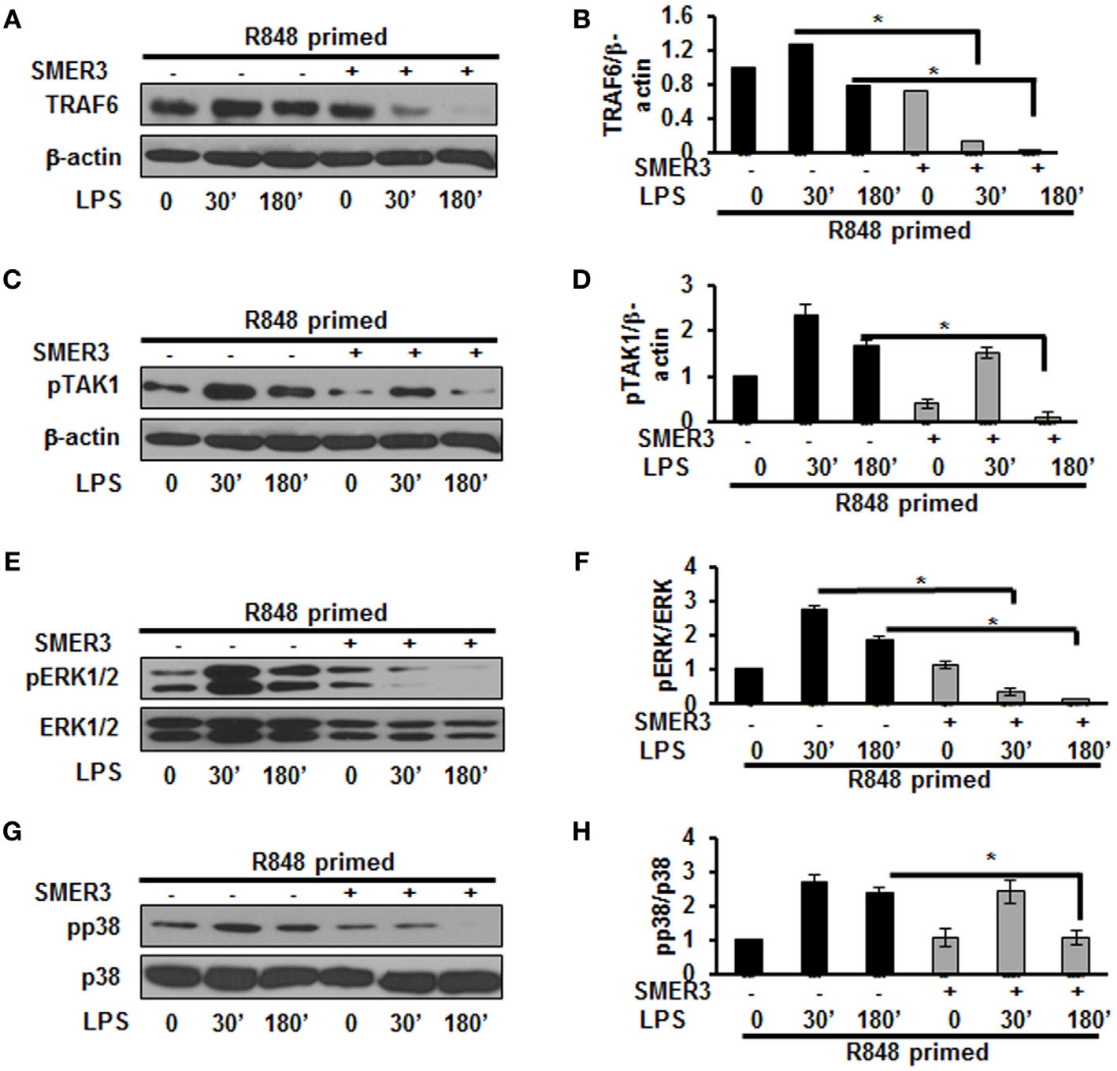
Small-molecule enhancer of rapamycin (SMER) 3 decreases MAPKs phosphorylation in R848-primed bone marrow-derived macrophages (BMDMs). BMDMs were preexposed to either R848 (100 ng/mL) or R848 and the E3 ubiquitin ligase inhibitor SMER3 (5 µM) for 24 h, cells were washed and subsequently challenged with LPS for different time periods. Whole cell extracts were subjected to SDS-PAGE and Western blot analysis performed using antibodies against TRAF6 and phosphorylated forms of TGF-β-activated kinase (TAK) 1, ERK1/2, and p38. TRAF6/β-actin or phosphorylated protein/total protein values of TRAF6 **(A,B)**, densitometry analysis expressed as fold increase of the ratio of phosphorylated TAK1 **(C,D)**, phosphorylated ERK1/2 **(E,F)**, and phosphorylated p38 **(G,H)**. Densitometry results shown are mean of five independent experiments. Representative results of Western blots are shown out of a total of five independent experiments. A *p* value <0.05 was considered significant and error bars indicate SEM.

Ubiquitination of proteins requires the participation of three enzymes: E1 ubiquitin activating enzyme, E2 ubiquitin-conjugating enzyme and an E3 ubiquitin ligase to attach ubiquitin molecules to a lysine of the substrate protein (26). TRAF6, a RING finger-containing protein, is an E3 ligase that plays an important role in the activation of the TLR pathway *via* K63-linked polyubiquitination of numerous signaling molecules (22, 23). As our results show that R848-primed BMDMs leads to an increased expression of K63-linked ubiquitinated proteins especially K63-linked ubiquitinated TRAF6, we determined the effect of SMER3, an E3 ULI, on TRAF6, the phosphorylation of TAK1, and the activation of MAPKs. SMER3 is a small-molecule ULI and a specific inhibitor of an SCF family E3 ubiquitin ligase that inhibits ubiquitination (27). This drug was non-toxic, as we found no changes in BMDMs viability in the presence of SMER3 as determined by MTT assay (Figure S2 in Supplementary Material). As shown in Figures 5A,B, SMER3 significantly downregulated TRAF6 expression after activation of primed cells with LPS at 30 min and 3 h. The mean densitometric values of the ratio of TRAF6/β-actin of five independent experiments are shown in Figure 5B. Similarly, there was a significant decrease in the phosphorylation of TAK1 in R848-primed BMDMs in response to LPS in the presence of SMER3 (Figure 5C). The mean densitometric values of the ratio of TAK1/β-actin of five independent experiments are shown in Figure 5D. Because SMER3 decreased TRAF6 expression and reduced phosphorylation of TAK1, we further elucidated the effect of SMER3 on ERK and p38 phosphorylation. SMER3 significantly inhibited phosphorylation of ERK1/2 at 30 min and 3 h in R848-primed cells followed by LPS challenge (Figure 5E). The mean densitometric values of the ratio of pERK/total ERK of five independent experiments are shown in Figure 5F. In addition, we observed a significant reduction of p38 phosphorylation in the presence of SMER3 in R848-primed cells after activation with LPS (Figure 5G). The mean densitometric values of the ratio of pp38/p38 of five independent experiments are shown in Figure 5H.

### SMER3 Reduces Polyubiquitination and Downregulates Excessive Cytokine Production in R848-Primed BMDMs in Response to LPS

**Figure 6:**
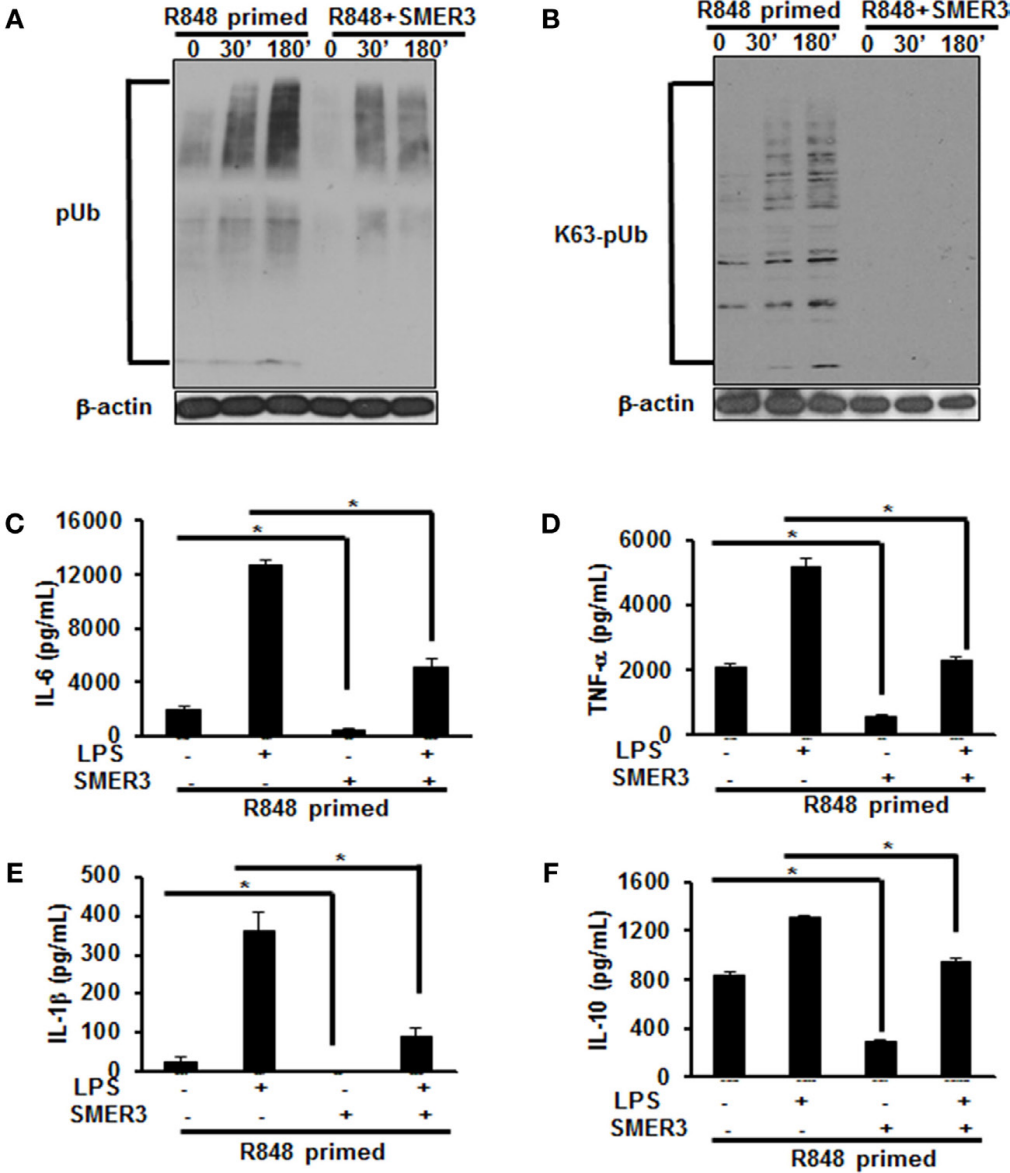
Pretreatment of R848-primed bone marrow-derived macrophages (BMDMs) with small-molecule enhancer of rapamycin (SMER) 3 reduces the expression of polyubiquitinated and K63-linked polyubiquitinated proteins and inhibits the production of inflammatory cytokines. BMDMs were preexposed to either R848 (100 ng/mL) or R848 and SMER3 (5 µM) for 24 h, cells were washed and subsequently challenged with LPS (500 ng/mL) for different time periods. Whole cell extracts were prepared and subjected to SDS-PAGE and Western blot analysis to assess the expression of polyubiquitin **(A)** and K63-linked polyubiquitin proteins **(B)**. Representative results of Western blot are shown out of a total of four independent experiments. Culture supernatants were collected after 24 h to measure IL-6 **(C)**, TNF-α **(D)**, IL-1β **(E)**, and IL-10 **(F)** production *via* enzyme-linked immunosorbent assay (ELISA). ELISA results shown are mean of four independent experiments. A *p* value <0.05 was considered significant, and error bars indicate SEM.

As priming of BMDMs with R848 increased polyubiquitination and specifically K63-linked polyubiquitination, we hypothesized that a drug inhibiting ubiquitin ligase such as SMER3 should reverse the effect of R848 priming. BMDMs were primed with R848 (100 ng/mL) in the presence or absence of SMER3 (5 µM). After the incubation period, cells were washed and challenged with LPS for different time periods. Whole cell extracts subjected to SDS-PAGE and Western blot analyzed for the expression of polyubiquitin as well as K63-linked polyubiquitinated proteins. Representative results of four independent experiments are shown in Figures 6A,B. SMER3 decreased the amount of polyubiquitinated proteins (Figure 6A) and K63 -linked polyubiquitinated proteins (Figure 6B) in R848-primed BMDMs. In addition, the presence of SMER3 inhibited LPS-mediated production of IL-6, TNF-α, IL-1β, and IL-10 (Figures 6C–F) significantly in R848-primed BMDMs. Data are presented as mean of four independent experiments and error bars indicate SEM. These results suggest that the inhibitory effect of SMER3 on MAPKs activation and production of pro-inflammatory cytokines is through inhibition of K63-linked polyubiquitination of proteins.

### R848 Priming of BMDMs Suppresses Bacterial Clearance and Increases Production of Pro-inflammatory Cytokines in Response to Live Bacteria

**Figure 7:**
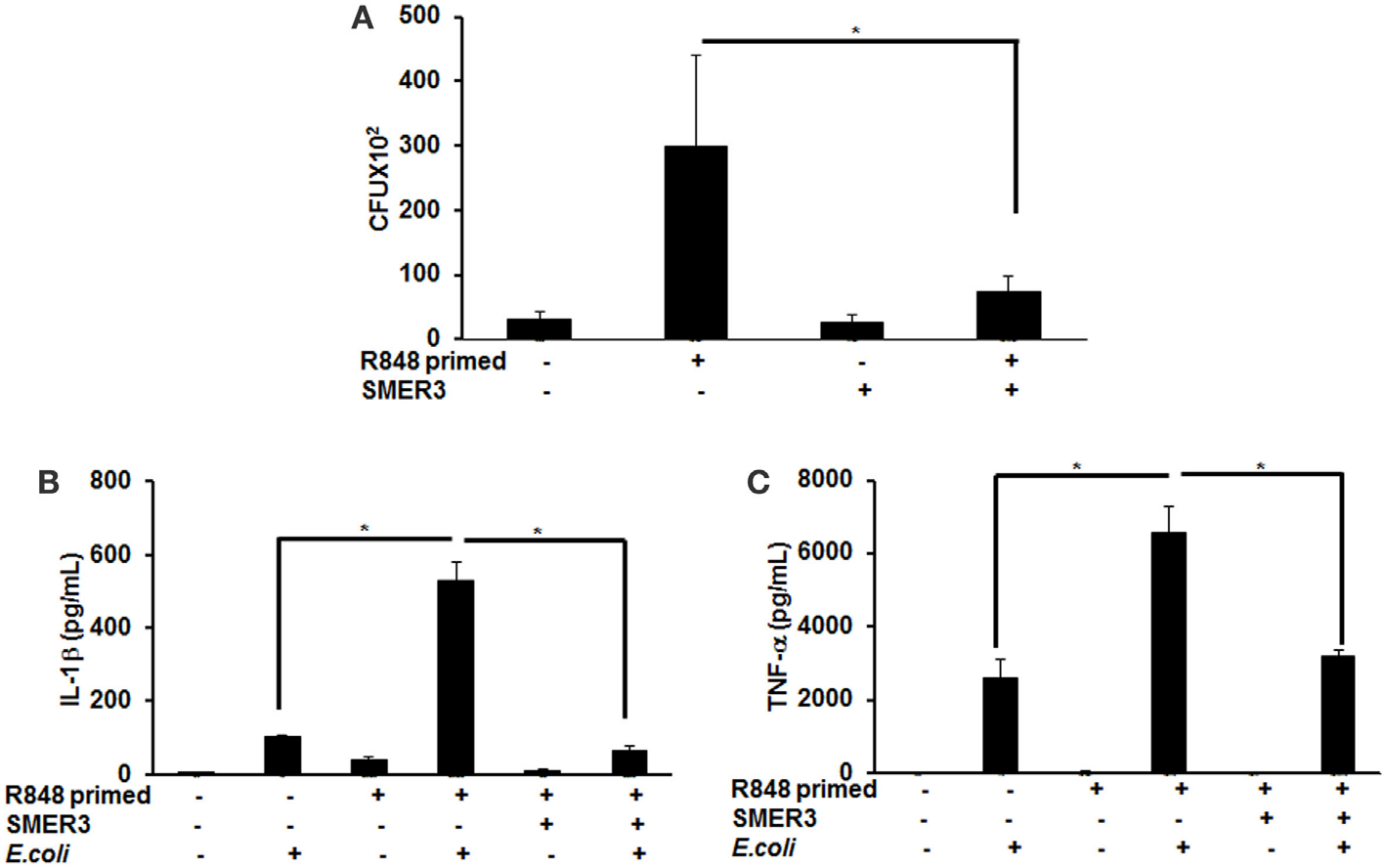
R848-primed bone marrow-derived macrophages (BMDMs) exhibit reduced bacterial clearance and produce significantly higher cytokines as compared with unprimed BMDMs on subsequent exposure to live *E. coli*. BMDMs were preexposed to either R848 (100 ng/mL) or R848 and small-molecule enhancer of rapamycin (SMER) 3 (5 µM) for 24 h, cells were washed and subsequently challenged with live bacteria *E. coli* with an MOI 10:1 (bacteria:BMDMs). Culture supernatants and cell lysates were collected 3 h after infection. For bacterial clearance assay, after 3 h of infection, BMDMs were lysed in PBS containing 0.2% TritonX-100, and a serial dilution of lysate was streaked in duplicate on LB agar plates, and incubated overnight at 37°C. CFUs were counted the next day. Data represent mean ± SEM from five different experiments and * represents *p* value <0.05 **(A)**. Culture supernatants were collected to measure IL-1β **(B)** and TNF-α **(C)**. Enzyme-linked immunosorbent assay results shown are mean of four independent experiments. A *p* value <0.05 was considered significant, and error bars indicate SEM.

To further investigate the effect of R848 priming on live bacterial infected BMDMs, we assessed bacterial clearance and cytokine production in response to *E. coli* infection on unprimed and R848-primed BMDMs. R848-primed BMDMs displayed a significantly decreased bacterial clearance as compared with unprimed BMDMs (Figure 7A). Pretreatment of BMDMs with SMER3 had no effect on bacterial clearance in unprimed BMDMs, whereas R848-primed BMDMs showed an increase in bacterial clearance (Figure 7A). Data are presented as mean of five independent experiments, and error bars indicate SEM. Finally, we determined cytokine production in response to *E. coli* infection in unprimed and R848-primed BMDMs. The infection of R848-primed BMDMs with live *E. coli* resulted in a significantly increased production of IL-1β (Figure 7B) and TNF-α (Figure 7C) as compared with unprimed BMDMs. SMER3 pretreatment of R848-primed BMDMs significantly decreased IL-1β and TNF-α production in response to bacterial infection (Figures 7B,C). Data are presented as mean of four independent experiments, and error bars indicate SEM.

## Discussion

Viral infections of the respiratory tract are often complicated by more serious bacterial infections (1). Secondary bacterial infections or coinfections after influenza illness are difficult to treat and are an important cause of morbidity and mortality worldwide (28, 29). While the influenza virus is the most common culprit in this context, other respiratory viruses, including respiratory syncytial virus, measles, parainfluenza virus, adenovirus, and rhinoviruses may also predispose a host to secondary bacterial infections (30, 31). The molecular basis of this predisposition is not well understood and the identification of the cellular mechanisms leading to synergy between viral and bacterial infections that offend host immune responses can be lifesaving. Various hypotheses exist and several mechanisms have been implicated including an immunosuppressive state in the host or hypo-activation of the immune system (32, 33). Other mechanisms suggested include an increased bacterial colonization and enhanced bacterial invasion (1), reduced neutrophil recruitment (34), post-viral desensitization to TLR signals (35), increased production of anti-inflammatory cytokines, especially IL-10 (36) and reduced phagocytic capacity of monocytes and macrophages (37). A recent study suggested that influenza infection leads to the depletion of alveolar macrophages, which are essential to the early clearance of bacteria (38). Interestingly, our data corroborate the recent data that post viral–bacterial infection results in a loss of immune tolerance and is associated with excessive inflammatory responses that may impair tissue repair (39).

Toll-like receptor 7 and TLR8 are intracellular TLRs that recognize nucleic acids following the internalization and lysing of single-stranded RNA viruses (27). TLR7/8 signals through a MyD88-dependent pathway that leads to NF-κB activation (28–30). Although single ligation of TLRs induces an inflammatory response, repeated exposure to the same TLR ligand results in an induction of immune tolerance (40). Several studies have shown that prior exposure of innate immune cells to low dose LPS (41–43), lipoteichoic acid from Gram-positive bacteria or the synthetic TLR2 ligand Pam3Cys (34) followed by the subsequent challenge with the same stimulus induces immune tolerance. In this study, we investigated whether the preexposure to a viral ligand (TLR7/8) induces priming or cross-tolerance in BMDMs on subsequent activation with bacterial ligands (TLR2 or TLR4). One previous study suggested that pretreatment with a MyD88-independent agonists (poly I:C) leads to a primed response to subsequent MyD88-specific agonists, whereas pretreatment with MyD88-specific agonists induces tolerance to one another (44). Our results demonstrate that preexposure of BMDMs to a TLR7/8 ligand does not induce cross-tolerance toward a subsequent challenge with TLR2 or TLR4 ligands. By contrast, preexposure of BMDMs to TLR7/8 ligand enhanced pro-inflammatory cytokine production after TLR2 or TLR4 challenge. In addition, R848 priming evoked a similar response to subsequent activation with TLR2 and TLR4 or live bacteria, suggesting a role of downstream players like TRAF6 rather than a MyD88-dependent mechanism. This notion is supported by our finding that TLR7/8 primed BMDMs exhibit upregulation of TRAF6 expression and increased TAK1 phosphorylation as compared with unprimed cells subsequently challenged with LPS or PAM. R848-primed cells exhibited increased phosphorylation of ERK1/2 and p38, whereas no difference was found in the phosphorylation of JNK between R848-primed and unprimed BMDMs. These data are in line with data from an *in vitro* model of post viral–bacterial infections published by Klemm et al., where JNK appears to have a minimal role in post viral–bacterial infection mediated IL-6 production (45). Importantly, we investigated whether the preexposure of murine macrophages to R848 alters bacterial clearance. We found that R848-primed macrophages have reduced bacterial clearance as compared with unprimed BMDMs. One of the mechanisms of reduced bacterial clearance in R848-primed macrophages might be due to decreased NO production. NO is bactericidal and is one of the important defense mechanisms for intracellular killing of bacteria (46, 47). In our model, the R848-primed BMDMs produced a lower amount of NO as compared with the unprimed BMDMs on subsequent challenge with LPS or PAM. In spite of excessive production of pro-inflammatory cytokines by R848-primed BMDMs in response to TLR2/4 as well as live bacteria, we observed a decreased bacterial clearance in R848-primed macrophages. These results confirm other studies that preexposure of macrophages with viral ligands may affect some of the innate immune mechanisms involved in pathogen clearance, including alteration of ROS, NO, or chemokines production and downregulation of scavenger receptors in macrophages (48–51). We speculate that preexposure of macrophages to viral ligands may downregulate scavenger receptors in the macrophages that are important for bacteria internalization or modify the ubiquitination machinery of macrophages important for bacterial killing. Further studies are needed to investigate the mechanisms involved in ROS, NO or chemokines production.

**Figure 8:**
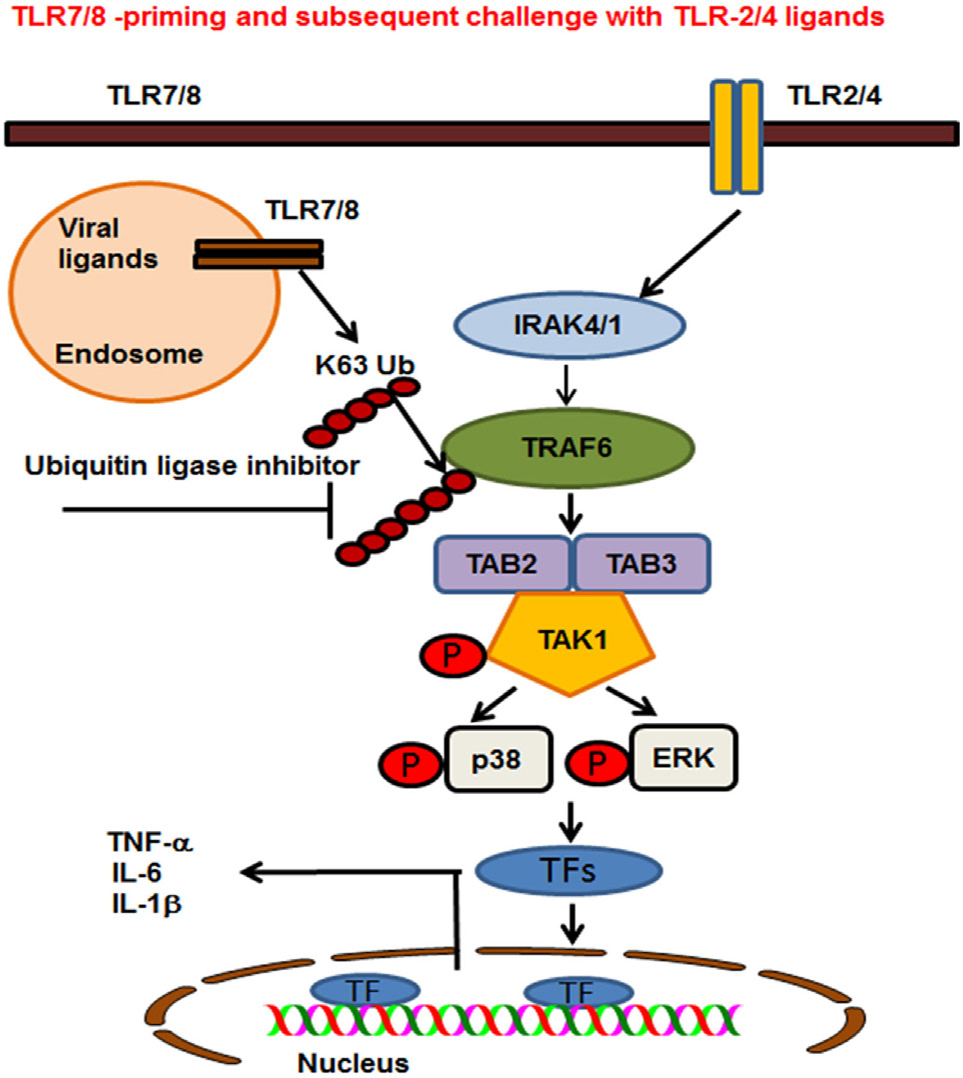
Proposed model of toll-like receptor (TLR) 7/8 priming of bone marrow-derived macrophages, increased activation of MAPKs, and production of inflammatory cytokines on subsequent exposure to TLR2/4 ligands. TLR7/8 is localized in the endosome where it recognizes ssRNA viruses or synthetic ligand R848. TLR2 and 4 are localized on the plasma membrane where they recognize the bacterial ligands PAM or LPS respectively. TLR7/8 priming leads to increased K63-linked polyubiquitination of proteins involved in TLR signaling and specifically TRAF6. Cells preexposed to viral ligands (TLR7/8) respond to subsequent stimulation with TLR2/4 ligands with K63-linked polyubiquitination of TRAF6, which leads to an enhanced activation of MAPKs and production of pro-inflammatory cytokines. Enhanced activation and cytokine storm are inhibited in the presence of an ubiquitin ligase inhibitor [small-molecule enhancer of rapamycin (SMER) 3]. SMER3 inhibits the K63 polyubiquitination of proteins, specifically the TRAF6 activity resulting in a decreased production of pro-inflammatory cytokines.

Ubiquitination plays an important role in regulating TLR signaling pathways (52). Ubiquitination controls the cellular processes *via* two modes of action. These are K48-linked ubiquitination of proteins that target substrates for proteasome degradation or K63-linked ubiquitination of regulatory proteins that provide scaffolding for the recruitment, assembly and activation of signaling complexes (53, 54). The findings of our study show that R848-primed BMDMs exhibit increased polyubiquitination and specifically K63-linked polyubiquitination as compared with unprimed BMDMs. By contrast, K48-ubiquitination was minimally affected in primed BMDMs (data not shown). Increased expression of K63-linked polyubiquitinated proteins was associated with TLR7/8 priming that may activate several proteins crucial in TLR signaling. Most importantly priming of BMDMs with TLR7/8 ligand upregulated the expression of K63-linked polyubiquitinated TRAF6, which was further increased upon challenge with LPS. K63-linked polyubiquitinated TRAF6 is an E3 ligase that auto-ubiquitinates itself and activates TAK1 and other TLR signaling molecules (10, 55). Other studies have reported that preexposure with TLR ligands followed by subsequent challenge with the same TLR stimulus induces a hypo-inflammatory state in the host, resulting in immune tolerance (56–59). In addition, these studies have shown that impaired activation of TLR signaling molecules including, IRAK-4, TAK1, and TRAF6, induces endotoxin tolerance (59). Increased expression of negative regulators of TLR signaling such as IRAK-M, SHIP-1, and A20 are reported to be associated with induction of endotoxin tolerance (41, 59). In the lights of these observations, we determined the expression of A20 in our model and found that LPS activation induced A20 in both R848-primed and unprimed cells (data not shown). This induction, though, was not effective to control hyperactivation of TLR signal transduction pathway in R848-primed BMDMs. The anti-inflammatory cytokine IL-10 has been shown to induce immunosuppression and tolerance (60). In our study, we found that although R848 priming induced significant production of IL-10, it failed to downregulate the production of pro-inflammatory cytokines. The results of the study by Berg et al. support our finding that IL-10 is not the main effector in inducing tolerance, but is the host’s natural defense against the development of a pathologic response to TLR ligands (61). Our findings indicate that although preexposure of BMDMs to TLR7/8 ligand results in the production of IL-10, an immunosuppressive cytokine, its induction could not downregulate pro-inflammatory cytokine production or had any effect on the TLR pathway and MAPKs activation. Based on these data, we hypothesized that inhibiting E3 ligase activity of TRAF6 may control an increased inflammatory response after LPS challenge. SMER3, a small-molecule E3 ULI, has been shown to be a specific inhibitor of a SCF family E3 ubiquitin ligase (27). We found that pretreatment of R848-primed BMDMs with SMER3 prevents R848 priming induced polyubiquitination, specifically the K63-linked polyubiquitination of proteins such as TRAF6 and phospho TAK1. This in turns results in decreased activation of MAPKs and reduction of pro-inflammatory cytokine production. Based on our results, we propose a model (Figure 8) that preexposure of a host to viral infections results in an increased expression of K63-linked polyubiquitinated proteins and TRAF6 K63-linked ubiquitination. K63-linked polyubiquitination on TRAF6 leads to increased assembly of K63-linked chains on downstream signaling molecules that result in phosphorylation of TAK1 and activation of MAP kinases. Subsequent exposure of viral infected BMDMs to bacterial ligand (TLR2/4) further leads to a synergistic production of inflammatory cytokines. Inhibition of K63-linked polyubiquitination on TRAF6 by SMER3 results in reduced activation of MAPKs and pro-inflammatory cytokine production. Our current study suggests that increased K63-linked polyubiquitination and activation of TRAF6 E3 ligase is one of the underlying mechanisms leading to the enhanced inflammatory responses seen in post viral–bacterial infections. Inhibition and modulation of E3 ligase activity seems to be a promising strategy to control post viral–bacterial infections and immune hyperresponsiveness. Thus, our study provides a new perspective in molecular targets to prevent cytokine storm and bacterial clearance after viral respiratory infections, as SMER3 reversed the effect of R848-primed BMDMs in response to TLR4 ligand.

## Author Contributions

JT contributed to the study design, conducted the experiments, and analyzed the data and in writing the manuscript. LS conceived and designed the study, participated in all areas of the research, data analysis and writing of the manuscript.

## Conflict of Interest Statement

The authors declare that the research was conducted in the absence of any commercial or financial relationships that could be construed as a potential conflict of interest.
