# Supplementary material for: K63-Linked Polyubiquitination on TRAF6 Regulates LPS-Mediated MAPK Activation, Cytokine Production, and Bacterial Clearance in Toll-Like Receptor 7/8 Primed Murine Macrophages
Source: Front Immunol. 2018 Feb 21;9:279. doi: 10.3389/fimmu.2018.00279 (PMC5826352; doi:10.3389/fimmu.2018.00279)
Supplement: Supplementary file 1 [file Data_Sheet_1.docx]

Supplementary Material

**K63-linked polyubiquitination on TRAF6 regulates LPS-mediated MAPK activation, cytokine production and bacterial clearance in TLR7/8 primed murine macrophages**

**Jaya Talreja^1^ and Lobelia Samavati ^1,2 *^**

^1^Department of Internal Medicine, Division of Pulmonary, Critical Care and Sleep Medicine, Wayne State University School of Medicine and Detroit Medical Center, Detroit, MI, USA;

^2^Center for Molecular Medicine and Genetics, Wayne State University School of Medicine, Detroit, MI, USA.

***Correspondence:**

Lobelia Samavati, MD

[ay6003@wayne.edu](mailto:ay6003@wayne.edu)

## Supplementary Figures

**Figure S1. Purity of BMDMs determined by flowcytometry**


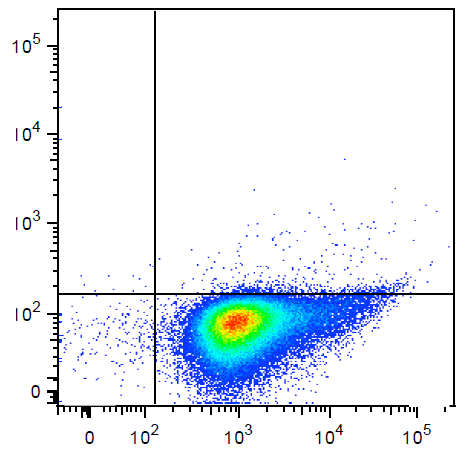


**CD11b**

**99.6%**

**SSC**

**Supplementary Figure 1.** C57BL6 wild type mice were used to isolate BMDMs. Briefly, femurs and tibias from 6- to 12-week-old mice were dissected, and the bone marrow was flushed out. Macrophages were cultured with IMDM media supplemented with 30% L929 conditioned media, glutamine, sodium pyruvate, 10% heat-inactivated fetal FBS, and antibiotics for 5-7 days. The purity of BMDMs was assessed by staining the cells with CD11b. The purity of BMDMs was found to be 99.6%.

**Figure S2. MTT assay to determine the toxicity of SMER3 on cell viability**

**Supplementary Figure 2.** Murine BMDMs were cultured at a density of 1x10^4^ cells/well in 96-well plate and were treated with LPS (500ng/ml), R848 (100 ng/mL) in the presence or absence of SMER3 (5μM) for 24h. Cell viability was assessed using MTT assay. Results represented are the mean ± SD of absorbance at 550nm of three experiments performed in triplicate.
